# Epidemiology of Coronavirus Disease 2019 in Japan: Descriptive Findings and Lessons Learned through Surveillance during the First Three Waves

**DOI:** 10.31662/jmaj.2021-0043

**Published:** 2021-07-09

**Authors:** Yuzo Arima, Kazuhiko Kanou, Takeshi Arashiro, Yura K Ko, Kanako Otani, Yuuki Tsuchihashi, Takuri Takahashi, Reiko Miyahara, Tomimasa Sunagawa, Motoi Suzuki

**Affiliations:** 1 Infectious Disease Surveillance Center, National Institute of Infectious Diseases, Tokyo, Japan

**Keywords:** SARS-CoV-2, surveillance, descriptive epidemiology, bias

## Abstract

**Introduction:**

Coronavirus disease 2019 (COVID-19) has caused unprecedented global morbidity and mortality. Japan has faced three epidemic “waves” of COVID-19 from early 2020 through early 2021. Here we narratively review the three waves in Japan, describe the key epidemiologic features of COVID-19, and discuss lessons learned.

**Methods:**

We assessed publicly available surveillance data, routine surveillance reports, and other relevant sources―multiple indicators were monitored to improve interpretation of surveillance data. Weekly trends for each wave were described based on the number of case notifications; number of tests performed; proportion of those tests that were positive for the novel coronavirus; the prevalent number of COVID-19 hospitalizations (total hospitalizations and those categorized as severe); and number of COVID-19 deaths. For each indicator and wave, we recorded the first calendar week to show an increase over two consecutive previous weeks, along with the peak week.

**Results:**

The spring wave was characterized by detection of cases imported from China, followed by notifications of sporadic cases without travel history, clusters, and mild/asymptomatic cases. The summer wave saw a large increase in notifications and a younger age distribution, but in the context of increased testing with lower test positivity. The winter wave brought considerable morbidity and mortality, surpassing the cumulative case counts and fatalities from the earlier waves, with high peak values. Overall, relative to the first wave, the burden of severe outcomes was lower in the second and higher in the third wave, but varied by prefecture. In all three waves, severe outcomes peaked after notification counts and test positivity peaked; severe outcomes were also consistently skewed toward the elderly.

**Conclusions:**

Important lessons were learned from each wave and across waves―some aspects remained constant, while others changed over time. In order to rapidly detect an increase in incidence, continuous, timely, and sensitive surveillance―using multiple information sources with careful interpretations―will be key in COVID-19 control.

## Introduction

A year has passed since the first case of coronavirus disease 2019 (COVID-19, the disease caused by severe acute respiratory syndrome coronavirus 2 [SARS-CoV-2]) was reported from Wuhan, China, rapidly evolving into a pandemic, unprecedented in scale and impact in modern history. Japan, with its high volume of travelers from China, strong reliance on public transport, dense urban cities, and aged population had been considered vulnerable, but has so far been spared of the morbidity and mortality experienced in some other countries^(1), (2)^. The country has, however, experienced its fair share of the pandemic’s impact, with cumulatively 386,370 laboratory-confirmed SARS-CoV-2 cases and 5,720 fatalities attributed to COVID-19 as at January 31, 2021^(3)^; and, similar to the rest of the world, the archipelago has to date faced three successive epidemic “waves,” with the third still ongoing as of February 2021.

Describing, characterizing, and assessing the basic epidemiology of a disease involves the time-honored attributes of “time,” “person,” and “place”^(4), (5)^. For infectious diseases such as COVID-19, information regarding the “virus” is also important given that genomic/phylogenic information can provide insights regarding the origin, disease transmission, or concerning genetic changes^(6), (7), (8)^. The concept of time is especially important in order to follow trends―for an acutely epidemic-prone emergent disease, it is imperative to monitor not only long-term trajectories but also short-term trends since the incidence can rapidly change. In addition, the distribution for “person,” “place,” and the “virus” can change over time, requiring stratified assessments and disaggregated summaries^(5)^.

Monitoring these key epidemiologic attributes in order to inform risk assessments and public health decisions necessitates surveillance. Here, a year into the pandemic, focusing on data from various surveillance systems (and other relevant sources of information), we narratively review the three epidemic waves in Japan, describe the key features of COVID-19, and discuss important lessons learned from surveillance.

## Materials and Methods

We used publicly available surveillance data shared by the Ministry of Health, Labour and Welfare websites^(3), (9), (10)^ to describe the trends and distributions of reported COVID-19 cases (includes symptomatic, presymptomatic, and asymptomatic SARS-CoV-2 infection cases). Supplementary information from subnational level government websites^(11), (12)^, routine surveillance reports ^(13), (14)^, and special studies/outbreak investigations ^(14), (15)^ were accessed for additional details and verification. We included data up to week 7 of 2021.

We summarized the three epidemic waves briefly, along with relevant contextual information. Temporal trends for each wave were described based on the number of confirmed case notifications, the number of laboratory tests performed, and the proportion of those tests that were positive for SARS-CoV-2 (i.e., test positivity; technically, a ratio given the data collection process). The incident number of deaths attributed to COVID-19 and the prevalent number of COVID-19 hospitalizations (total hospitalized and those categorized as severe) were also described. As the number of currently hospitalized cases were reported on a daily basis (i.e., a prevalent measure based on the net result of case-patients already hospitalized plus new hospitalizations minus discharges and deaths), for hospitalization-based indicators, the weekly mean of the daily number of prevalent cases was monitored. For each indicator and wave, we recorded the first calendar week to show an increase in the indicator’s value over two consecutive previous weeks (i.e., the indicator’s value for week X is greater than that for week X-1, and that for week X-1 is greater than that for week X-2)^(16)^, along with the week with the maximum value (i.e., peak week). Weeks were based on the Monday-Sunday schedule^(17)^.

We monitored multiple indicators other than confirmed case counts to reduce misinterpretations due to surveillance biases and enhance the confidence level in situational awareness ^(18), (19), (20), (21), (22), (23), (24)^. The numbers of tests performed and the test positivity were assessed to account for test intensity (to consider the frequency of confirmed cases given the number of tests performed). The number of deaths and hospitalizations were assessed to monitor a restricted subset of cases that would be less affected by changes in testing intensity over time and also in health-seeking behaviors (since clinically more ill case-patients were initially targeted for testing and such patients would also be more likely to seek care, regardless of time or place).

## Results

### The first wave

Soon after the first COVID-19 case was reported in December 2019 from China, the first confirmed case in Japan was reported on January 15, 2020^(13), (25)^. The patient presented to a medical facility with pneumonia, and with recent travel history to Wuhan, was suspected and notified through the National Epidemiological Surveillance of Infectious Diseases surveillance system^(13), (26)^ as the first official COVID-19 case in Japan. Reports of additional detections followed, not only from those who sought care with respiratory symptoms but also from those that were presymptomatic/asymptomatic or with only mild clinical signs/symptoms; many such infections from the milder clinical spectrum were detected from persons repatriated from Wuhan via chartered flights (approximately 800 individuals) and from crew members and passengers on the Diamond Princess cruise ship (approximately 3,700 individuals)^(27), (28), (29)^. For severe outcomes, the elderly appeared to be particularly at risk^(3)^.

**Figure 1.**
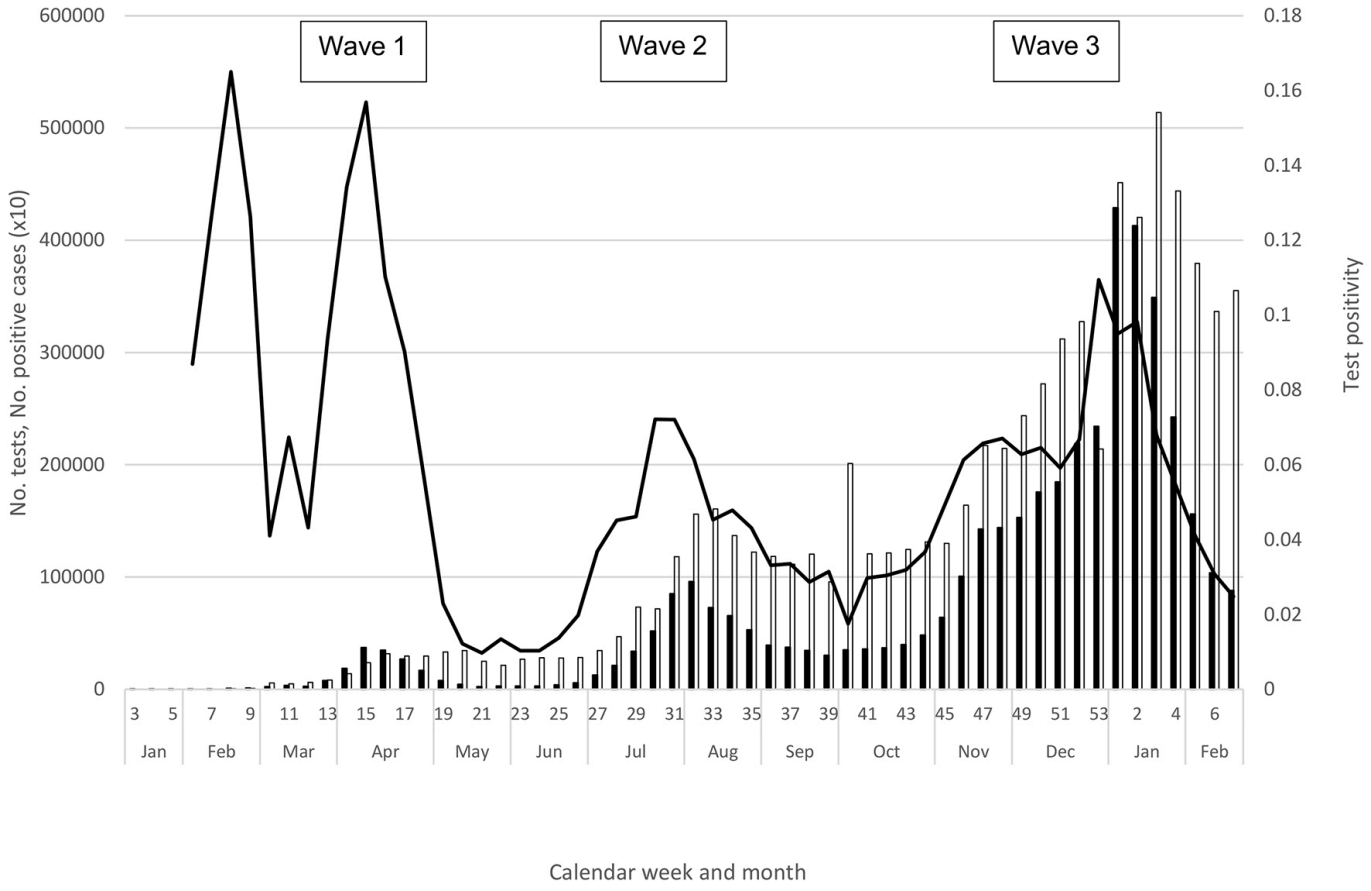
Number of SARS-CoV-2-positive cases (black bars), number of SARS-CoV-2 tests (white bars), and test positivity (number of SARS-CoV-2-positive cases divided by number of SARS-CoV-2 tests, black line), by week, week 3, 2020 to week 7, 2021, Japan. Number of SARS-CoV-2-positive cases is multiplied by 10 for easier viewing. Week 40 includes delayed batch reporting of number of tests (https://www.mhlw.go.jp/stf/covid-19/open-data.html).

**Figure 2.**
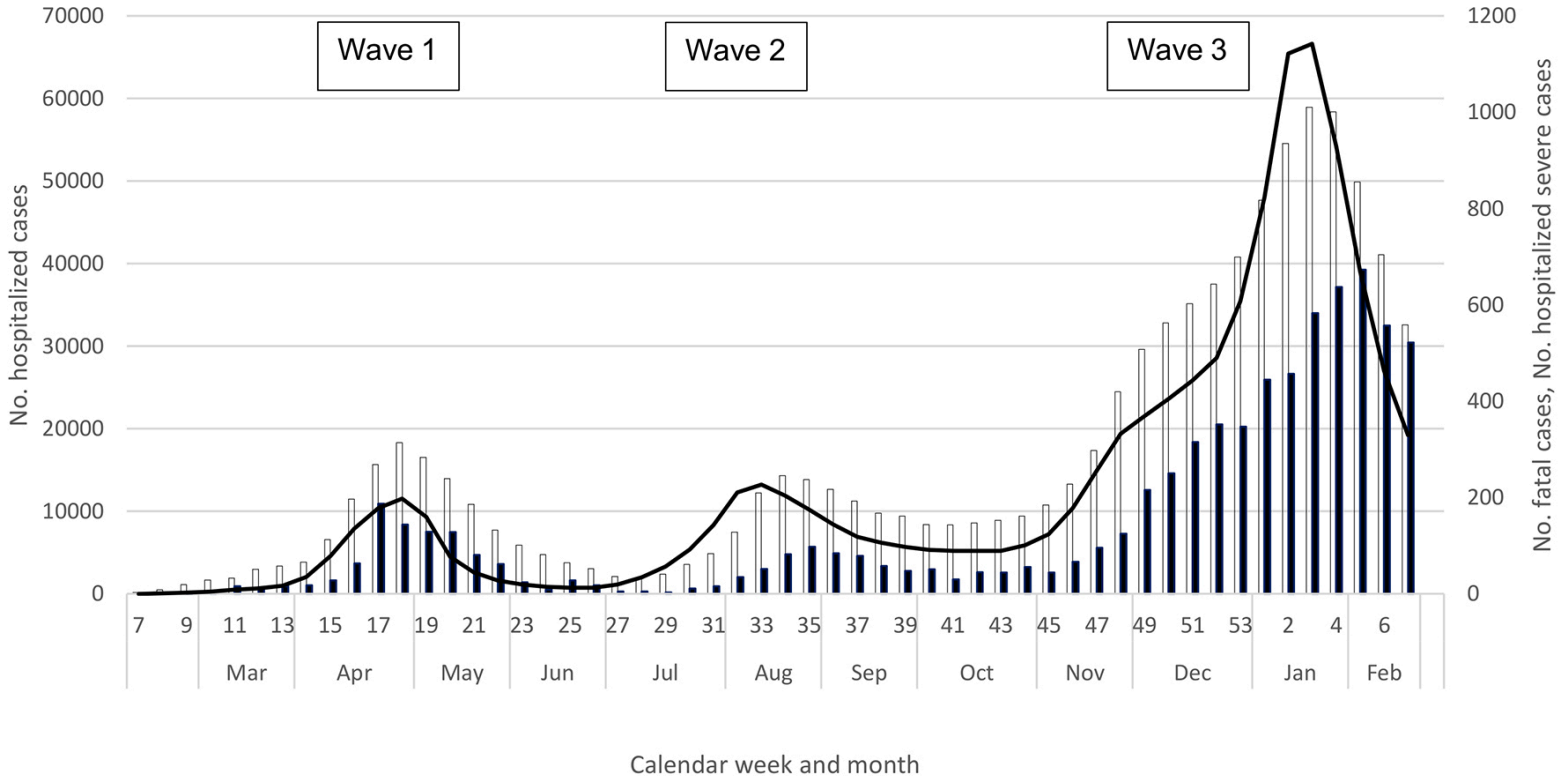
Number of COVID-19 deaths (black bars), weekly average number of prevalent hospitalized severe COVID-19 cases (white bars), and weekly average number of prevalent hospitalized COVID-19 cases (black line), by week, week 7, 2020 to week 7, 2021, Japan. Week 17 includes delayed batch reporting of COVID-19 deaths (https://www.mhlw.go.jp/stf/covid-19/open-data.html).

Through March, the number of confirmed cases, number of tests, and positivity―along with prevalent hospitalized cases, prevalent hospitalized severe cases, and deaths―all continued to increase (Table 1, Figure 1 and 2); this increase occurred despite the official closure of all schools through the month of March. Initially, the majority of cases were imported, but those that did not report an overseas travel history that were “sporadic” (i.e., without an epidemiological link to another case) increased^(13), (30)^. In addition, numerous clusters of cases began to be reported from metropolitan areas such as Tokyo and Osaka prefectures^(13), (30), (31)^. Molecular epidemiologic analyses indicated that the first wave was actually composed of two waves―the earlier series of cases in January and February were attributable to the virus from Wuhan, while cases in March and April were infected with the viral strain that was circulating in Europe^(6)^. With the rapid rise in case notifications and the increasingly burdened medical and public health sectors, the Japanese government issued an emergency declaration to seven urban prefectures on April 7, and a nationwide declaration on April 16. Fortunately, likely attributed to a reduced contact rate between individuals (as suggested from a steep decline in human mobility data^(32)^), all indicators except for the number of tests performed peaked in April and steadily declined thereafter (Table 1, Figure 1 and 2), and the state of emergency was declared over in late May.

**Table 1.** First Week to Show an Increase Over Two Consecutive Previous Weeks and Peak Week for Key Surveillance Indicators of COVID-19, by COVID-19 Epidemic Wave, Japan, from Week 8, 2020 to Week 7, 2021 (as at February 28, 2021)^a^.

	1) No. positive cases	2) No. tests	3) Test positivity	4) Average no. hospitalized cases	5) Average no. hospitalized severe cases	6) No. deaths
**First wave**						
First week with increase over two consecutive weeks*	8	8	8	8	8	14
Peak week	15	20	8, 15	18	18	18**
**Second wave**						
First week with increase over two consecutive weeks	25	24	25	27	30	31
Peak week	32	33	30, 31	33	34	35
**Third wave**						
First week with increase over two consecutive weeks	41	43***	42	45	43	47
Peak week	1	3	53	3	3	5

^a^ the first week to show an increase over two consecutive previous weeks is when the indicator’s value for week X is greater than that for week X-1, and that for week X-1 is greater than that for week X-2. Surveillance indicators are 1) the number of SARS-CoV-2 positive cases; 2) the number of SARS-CoV-2 tests; 3) test positivity (number of SARS-CoV-2 positive cases divided by number of SARS-CoV-2 tests); 4) weekly average number of prevalent hospitalized COVID-19 cases; 5) weekly average number of prevalent hospitalized severe COVID-19 cases; and 6) number of COVID-19 deaths. All weeks are calendar weeks as defined by https://www.niid.go.jp/niid/ja/calendar.html. *data available from week 6. **excludes batch report in week 17 (https://www.mhlw.go.jp/stf/covid-19/open-data.html) ***excludes batch report in week 40 (https://www.mhlw.go.jp/stf/covid-19/open-data.html)

### The second wave

The decline in indicators was short-lived; the numbers of notifications, tests, and test positivity began to rise again in June (Table 1 and Figure 1), and the hospitalization indicators and deaths increased in July (Table 1 and Figure 2). In fact, notifications and clusters were already beginning to increase in parts of Tokyo Prefecture in late May, and Tokyo was seeing a rise in the numbers of notifications, tests, and test positivity from late May ^(11)^―even though international travel restrictions remained and domestic travel was still limited for the Kanto Region and Tokyo residents. Fortunately for Tokyo, while there were more notifications in the summer, the prefecture experienced considerably fewer severe cases than in the spring ^(11)^. While this trend was also observed nationally overall (Figure 2), the western metropolis Osaka faced a similar burden of severe cases during the first and second waves, and Aichi and Okinawa prefectures experienced considerably more severe cases than in the spring ^(10), (12)^, with both declaring a prefectural state of emergency in August. Similar to the spring, however, the overall COVID-19 burden was lower in rural prefectures with a low population density ^(3), (9), (10)^. Nationally, all indicators increased in July, peaked during late July through August, declined in September, and plateaued through the first half of October; notably, case notifications and hospitalizations never declined to the levels in May (Table 1, Figure 1 and 2).

A notable change during summer 2020 was the substantial increase in tests ^(3), (10)^, likely leading to increased ascertainment of milder cases, who were more likely to be young. In fact, while notifications and tests both increased in the second wave, test positivity declined substantially in the summer (Figure 1). Another change during the summer was the lower COVID-19 mortality (Figure 2; mortality remained lower even if including the number of deaths one month after the end of each wave to account for the time lag in the occurrence of deaths). Importantly, however, severe and fatal cases continued to be skewed toward the elderly, particularly affecting older men ^(3), (9), (10)^.

### The third wave

Within the early autumn backdrop of steady notifications and relatively high prevalence of hospitalized case-patients, cases again began to rise in the latter half of October. Still ongoing as of early 2021, the third wave has resulted in substantial COVID-19 morbidity and mortality, surpassing both the total and fatal case counts from the first two waves combined (Figure 1 and 2). In October, notifications, tests, and test positivity all increased (weeks 41-43), along with hospitalized cases, and all indicators continued to increase through December (Table 1, Figure 1 and 2). By early December, the weekly number of fatalities surpassed the respective peaks from the earlier waves (Figure 2). Although the magnitude of the increase varied, resurgence was observed nationwide, in urban and rural regions, from Hokkaido in the north (where the impact was particularly serious) to Okinawa in the south ^(3), (9), (10)^. “Go To Eat” (initiated in late September 2020 to encourage dining out) was stopped in late November ^(33)^, while “Go To Travel” (introduced to encourage domestic travel, from October 2020 for Tokyo and July 2020 for other prefectures) was halted in late December ^(34)^. In early December, Osaka Prefecture declared its own medical state of emergency, and the Japan Medical Association declared a medical state of emergency in late December. A state of emergency was declared for the Kanto prefectures in early January 2021, followed by several other prefectures.

As at February 28, 2021, all indicators appeared to have peaked, ranging from week 53 of 2020 (test positivity) to week 5 of 2021 (deaths) (Table 1), with a decline thereafter, albeit more gradual for deaths (Figure 1 and 2). As in the earlier waves, the respective peaks for number of prevalent hospitalizations, prevalent severe cases, and incident deaths lagged behind those for notifications and test positivity (Table 1); moreover, fatal cases continued to be skewed toward the elderly ^(3), (9), (10)^.

## Discussion

Important insights and lessons regarding COVID-19 were learned from each wave and across the successive waves. Early on from the first wave, key features about COVID-19 epidemiology in general and those specific to Japan became apparent. First, the cohorts from the chartered flights from Wuhan and the Diamond Princess cruise ship provided early empirical evidence that the “severity pyramid” for SARS-CoV-2 infection ^(35), (36)^ includes a sizable proportion of subclinical and asymptomatic infections. Nearly a quarter of the cases from the Wuhan returnee cohorts were, in fact, asymptomatic^(27), (37)^. Such insights could be gained because all members from these cohorts underwent polymerase chain reaction testing, regardless of sign/symptom presence, with many of them having close follow-up for disease onset and subsequent testing at the end of the observation period. Such universal testing schemes filled in the early knowledge gaps that could not be obtained from routine surveillance ^(27)^―where the likelihood of testing was indicated by the presence and/or severity of clinical presentation, and systematic follow-up to determine whether persons were asymptomatic or presymptomatic at the time of testing was lacking. While the substantial proportion of asymptomatic infections has since been acknowledged ^(38)^, these early data also served as an important reminder that the clinical spectrum of case-patients reported through surveillance represent a non-representative sample of SARS-CoV-2-infections that sought care and were selectively suspected, tested, and reported.

As with other recent infectious disease outbreaks both domestic and overseas ^(39), (40)^, molecular epidemiology proved valuable from the early stage of the pandemic, providing insights not possible from conventional epidemiology ^(41)^. Genomic sequencing showed that the first wave was actually composed of two different waves (Wuhan/China and European) ^(6)^, indicating that the earlier spread from cases imported from China was limited, with much of the COVID-19 burden in the spring associated with cases imported from Europe and subsequent transmission from those cases. Such discrimination from genomic surveillance provided a more confident assessment that the early cases imported from China had not led to widespread but undetected transmission in Japan, and that the lack of an increase in notifications (albeit an underestimate of the true incidence) was not simply a surveillance artifact; subsequent seroprevalence surveys confirmed the extremely low prevalence of infections in Japan ^(42)^. With the advantage of being an island nation, Japan―along with Taiwan, New Zealand, and Australia―presented real-world examples that containing and/or controlling the virus were possible if effective public health measures were implemented. Molecular epidemiology and virological surveillance have continued to provide useful intelligence ^(8), (43), (44)^, with the recent variants of concern illustrating its particularly significant role ^(7)^.

While these early findings indicated that not all those infected would have a severe outcome and that the epidemic was controllable, the experience in April demonstrated that COVID-19 can be acutely epidemic-prone in Japan (Figure 1 and 2), which was hitherto spared of both the severe acute respiratory syndrome pandemic and Middle East respiratory syndrome outbreaks. Observed globally since then, the epidemiology of COVID-19 was such that once SARS-CoV-2 circulates in the community, the incidence can surge with a rapid rise in severe cases that can overwhelm hospitals. This presented a threat in populous settings such as Tokyo due to the high volume of COVID-19 patients; given the limited medical capacity, rural regions also faced a challenge even with a low absolute number of cases. From a surveillance perspective, the potential for such a surge meant that rapid decision-making to initiate timely response was necessary, which in turn meant that monitoring frequently over a short interval―ideally daily―was important. This was challenging because the quantity and quality of information to be collected had to be balanced with the timeliness of reporting, when healthcare workers were already stretched thin.

Assessing the data by time, place, and person, the second wave provided additional lessons. First, depending on the prefecture, the COVID-19 experience in the second wave proved to be different from the first wave―some experienced more, others fewer, severe cases than in the spring. While the first wave had a strong impact on Tokyo, the second wave had fewer severe cases and deaths despite increased notifications ^(11)^. In contrast, other prefectures such as Osaka and Okinawa faced a very different summer, with hospitalization burden equal to or greater than that of the first wave ^(10), (12)^. Such place-dependent heterogeneity over time reinforced the importance of continuous and vigilant surveillance, as past waves may not be predictive of future trends or distributions.

However, some aspects pertaining to “place” remained constant. On average, rural prefectures with a low population density (e.g., some prefectures in the Tohoku, Sanin, and Shikoku regions) had lower notification rates during both waves relative to more populated prefectures (e.g., prefectures in the Kanto and Kansai regions) ^(3), (10)^. Moreover, regardless of prefecture, community transmission was often linked to urban centers with dining and entertainment venues^(13), (14)^. Such consistencies helped to enhance monitoring in certain locations and populations, and to target preventive interventions and risk communications.

A similar observation during the second wave was made for the “person” attribute. In the second wave, while there was a shift to a younger age distribution among the reported cases, severe/fatal cases continued to be skewed toward the elderly, especially older males ^(3), (9), (10), (45), (46)^. This pattern was reported from other regions/countries, across various race/ethnicities ^(1), (47)^. During the first wave, part of the reason that cases were older was likely due to testing strategies targeting those that were severe or indicative of potentially severe outcomes (i.e., older age). Despite a substantial increase in testing over the summer months, with many younger individuals receiving the test, severe case-patients were still disproportionately old ^(3), (9)^.

While enhanced ascertainment for milder cases likely lowered the age distribution, there seemed to be some indication that the younger demographic had a higher incidence during the summer. There was an increase in clusters at entertainment clubs/lounges ^(3), (13)^, often captured by event-based surveillance (EBS) which was believed to have a similar level of sensitivity during the first and second waves. The shift to a younger age group was also noted in Europe and the US, where testing intensity was considerably higher than Japan from the spring ^(2)^; in the US, increased notifications in younger age groups were accompanied by increased testing and positivity, suggesting that enhanced ascertainment alone was unlikely to explain the increased notification rate in these groups ^(48), (49)^.

Regardless of the reason(s) of the increase in notifications of younger cases (socio-behavioral, biological, and/or surveillance-related), the second wave appeared to have a lower impact on the elderly―despite increased case notifications, the absolute numbers of hospitalizations and deaths were actually lower in the second wave (Figure 2). While COVID-19 mortality in the Japanese population declined―and the reason(s) for this decrease is beyond the scope of this discussion (e.g., improved treatment/care, change in the virus’s virulence, change in exposure-related behaviors, a “harvesting effect,” reduction in nursing home outbreaks)―the take-home message was that those at high risk of severe outcomes remained unchanged, allowing clinicians and public health workers to provide consistent messages to protect the most vulnerable.

Lastly, while still ongoing, the third wave has reaffirmed some of the lessons learned from the earlier waves. Some trends and distributions remained constant―the elderly (particularly older men) continued to be associated with severe outcomes ^(3), (9), (10)^, with more urban areas on average experiencing a higher absolute and per-population burden ^(3), (9), (10)^. In all three waves, severe outcomes peaked consistently later than those for notifications and test positivity, demonstrating a well-known lag effect ^(47), (50)^. However, some aspects were time-variant―hospitalizations for severe case-patients returned to high levels in Tokyo and dramatically increased in the Kansai region (and in some other prefectures such as Hokkaido, Tochigi, Gifu, and Saitama), whereas Okinawa fortunately faced a lower burden in the third wave ^(3), (9), (10)^.

Perhaps most importantly, almost a year since the first wave, the third wave was a stark reminder that COVID-19 can be intensely epidemic-prone. Causing substantial morbidity and mortality over a span of just few weeks, the third wave has already surpassed the cumulative case counts and fatalities from the first two waves. Beginning in October, case notifications and positivity increased, followed by a rise in hospitalizations and deaths (Table 1, Figure 1 and 2). Contrary to the second wave, which saw increased testing but a decline in positivity over the first wave, the third wave saw positivity increase in the context of considerably increased testing over the second wave. In addition, the third wave saw a large increase in severe outcomes (Figure 2)―taken together, these data suggested a true increase in incidence and that enhanced ascertainment was unlikely to explain the rise in COVID-19 notifications during the third wave. Similar trends of increased positivity, hospitalizations, and deaths were reported from other temperate countries in the northern hemisphere in the winter ^(1), (2)^, with great strain on the healthcare and economic sectors.

Thus, from a surveillance perspective, the pandemic reaffirmed the importance of accounting for testing intensity and severe outcomes, and the danger of direct comparisons of notification rates as “incidence” over time or place ^(18), (21)^. Interpreting the sometimes-changing epidemiology of COVID-19 from surveillance can be challenging, and as we continue to monitor COVID-19, a pluralistic approach with multiple information sources and approaches will be critical. This approach allows us to sensitively detect early warning signs and a potential increase in incidence; in addition to the indicators presented here, other supplementary indicators can be monitored, such as reports of clusters via EBS ^(19)^ and syndromic approaches such as fever hotline calls ^(11)^. Monitoring multiple data sources and indicators also helps prevent false alarms (e.g., increase in notifications due to increased testing, batch reporting), thereby improving the specificity of assessments and subsequent decisions.

With only a year of experience with SARS-CoV-2, we should be cautious of generalizations and being overly confident in predictions and assumptions based on past experience or other coronaviruses―we have yet to understand annual trends and seasonality, and vaccinations may modify the epidemiology of COVID-19. We have also shown that aggregate summaries can mask important heterogeneities and that trends, magnitudes, and distributions can change over time and differ by person, place, or virus. A continuous, timely, and sensitive surveillance strategy―making use of multiple information sources and approaches with careful interpretations―will be key in controlling this evolving virus.

## Article Information

### Conflicts of Interest

None

### Acknowledgement

The authors are thankful to all the clinicians and staff members of local public health centers and prefectural and municipal public health institutes for engaging in surveillance, assessment, and response activities in Japan. The authors also acknowledge the data collection, collation, and feedback performed by members of the Ministry of Health, Labour and Welfare and the numerous volunteer staff.

### Author Contributions

All authors participated in conception, analysis, and/or interpretation of the data; drafting the article; or revising it critically. All authors approved the final version.

### Approval by Institutional Review Board (IRB)

NA. No ethical approval was necessary because the descriptions were conducted for public health purposes using publicly available national surveillance data and information collected in compliance with the Infectious Diseases Control Law.
